# The bHLH-PAS Transcription Factor Dysfusion Regulates Tarsal Joint Formation in Response to Notch Activity during *Drosophila* Leg Development

**DOI:** 10.1371/journal.pgen.1004621

**Published:** 2014-10-16

**Authors:** Sergio Córdoba, Carlos Estella

**Affiliations:** Departamento de Biología Molecular and Centro de Biología Molecular Severo Ochoa, Universidad Autónoma de Madrid (UAM), Madrid, Spain; New York University, United States of America

## Abstract

A characteristic of all arthropods is the presence of flexible structures called joints that connect all leg segments. *Drosophila* legs include two types of joints: the proximal or “true” joints that are motile due to the presence of muscle attachment and the distal joints that lack musculature. These joints are not only morphologically, functionally and evolutionarily different, but also the morphogenetic program that forms them is distinct. Development of both proximal and distal joints requires Notch activity; however, it is still unknown how this pathway can control the development of such homologous although distinct structures. Here we show that the bHLH-PAS transcription factor encoded by the gene *dysfusion* (*dys*), is expressed and absolutely required for tarsal joint development while it is dispensable for proximal joints. In the presumptive tarsal joints, Dys regulates the expression of the pro-apoptotic genes *reaper* and *head involution defective* and the expression of the RhoGTPases modulators, *RhoGEf2* and *RhoGap71E*, thus directing key morphogenetic events required for tarsal joint development. When ectopically expressed, *dys* is able to induce some aspects of the morphogenetic program necessary for distal joint development such as fold formation and programmed cell death. This novel Dys function depends on its obligated partner Tango to activate the transcription of target genes. We also identified a dedicated *dys cis*-regulatory module that regulates *dys* expression in the tarsal presumptive leg joints through direct Su(H) binding. All these data place *dys* as a key player downstream of Notch, directing distal *versus* proximal joint morphogenesis.

## Author Summary

One of the keys of the evolutionary success of arthropods, the most diversified group of animals, is the acquisition of joints that allow the articulation of their appendages. Two main kinds of joints with different morphologies and evolutionary origin are found in the fly leg: the proximal or “true” joints that are motile due to muscular attachment and the distal joints that are immotile. A common event during joint formation is the activation of the Notch pathway at the presumptive joints along the leg proximo-distal axis. In this work we investigated how the same pathway, Notch, can control the formation of such homologous although different structures. We described that the transcription factor Dysfusion (Dys) is a Notch target required for distal joint development and that is sufficient to induce joint-like structures when ectopically expressed. Dys controls two important morphogenetic events that direct tarsal joint development such as programed cell death and epithelial cell shape. Moreover, we identified a regulatory DNA sequence that controls *dys* expression in the tarsal segment by direct binding of the transcriptional effector of the Notch pathway Su(H). Thus, Notch controls the development of proximal *vs* distal joints by the recruitment of specific downstream target genes such as *dys*.

## Introduction

Throughout evolution, animal appendages have diversified to display very different morphologies, which are indicative of their diverse functionality such as locomotion, feeding or environment exploration. One of the keys of the evolutionary success of arthropods (from Greek *árthron*, “joint”, and *pous* i.e. “foot”), the most diversified group of animals, is the acquisition of joints that allow the articulation of their appendages. Appendages are external projections from the body wall, which require the formation of a proximo-distal axis (PD) that is specified *de novo* during development from previously established antero-posterior and dorso-ventral axes. In *Drosophila*, thoracic appendage primordia are specified during embryogenesis by the expression of the homeobox gene *Distalless* (*Dll*) [1]. During larval development, the PD axis is generated by the juxtaposition of cells that express two signaling molecules, *wingless* (*wg*) and *decapentaplegic* (*dpp*). They regulate the expression of the leg “gap genes” *Dll*, *dachshund* (*dac*) and *homothorax* (*hth*) dividing the leg into distal, medial and proximal domains, respectively (reviewed by [2]) [3]–[8]. Subsequently, the distal leg is further subdivided into more discrete domains of tarsal-specific gene expression in response to the Epidermal Growth Factor Receptor (EGFR) pathway activity [9], [10].

The *Drosophila* adult leg is divided in 10 segments (coxa, throchanter, femur, tibia, five tarsal segments and the claw or pretarsus), which are articulated due to the presence of joints in between them. A common event during joint formation is the positioning of Notch ligands Delta (Dl) and Serrate (Ser) by the combined action of the leg “gap genes” and PD tarsal genes in concentric rings at the proximal end of each segment [11]–[14]. Notch pathway activation adjacent and distal to the *Dl* and *Ser* expression domains involves the proteolytic cleavage and release of the intracellular fragment of Notch (NICD) that acts as a transcriptional co-activator with proteins of the CBF1-Suppressor of Hairless (Su(H)) family [15]. In the leg joints, Notch pathway activation, visualized by the expression of its target genes *Enhancer-of-split mβ* (*E(spl)mβ*) and *big brain* (*bib*), mediate leg segmentation and growth. Therefore, mutant clones for components of the Notch signaling pathway that span two leg segments are associated with joint and growth defects [12], [14], [16], [17].

While Notch activity is absolutely required for all joints, proximal and distal joints are functionally, morphologically and evolutionarily different [18]–[24]. Proximal joints, also known as “true joints”, such as the tibial/tarsal joint, are asymmetrical and motile due to muscular attachments. In contrast, distal joints, the ones that separate the tarsal segments, are radially symmetrical, not motile and depend on a different molecular mechanism for their development [20], [22]. Moreover, several genes are specifically expressed in the “true joints” such as the *odd-skipped* (*odd*) family members *odd*, *drumstick* (*drm*) and *sister of odd and bowl* (*sob*), and others are restricted to the tarsal ones, like *deadpan* (*dpn*) or *tarsal-less* (*tal*) [14], [25], [26].

Two morphogenetic processes, apoptosis and changes in cell shape, contribute to joint formation [20], [27]. Apoptosis in the legs involves the Jun kinase (JNK)-mediated activation of the pro-apoptotic gene *reaper* (*rpr*) in response to sharp boundaries of Dpp activity [20], [28]. The early expression of *dpp* in the leg disc in dorsal-anterior cells is later refined into a segmented pattern of incomplete rings with higher levels at the distal edge of each segment [20]. It has been suggested that a confrontation of cells with different levels of Dpp pathway activity at the distal end of tarsal segments triggers cell death via JNK activation at both sides of the activity discontinuity [20], [28]. Interestingly, the cell death-mediated mechanism is required for joint architecture only in the tarsal segments [20]. Changes in cell shape are in part mediated by the modulation of RhoGTPases activity [27]. RhoGTPases function as molecular switches (active GTP-bound state and inactive GDP-bound state) that regulate a variety of developmental processes such as cytoskeletal dynamics, cell migration, cell polarity, cell-cycle progression, vesicle trafficking and cytokinesis [29]. A subset of RhoGEFs and RhoGAPs, proteins that regulate Rho activity, are specifically expressed in all joints or restricted to tarsal joints, and the downregulation of some of them produce defects in leg joint formation [27].

An important and yet unresolved question is to understand how the same signaling pathway, Notch, could direct the formation of homologous but morphogenetically distinct structures, such as the different joints along the PD axis of the leg, which will require the deployment of different genetic programs for their formation [11], [12], [14], [20], [22]. In this work we characterize the expression, function and regulation of the Npas4/NXF vertebrate ortholog *dysfusion* (*dys*). *dys* encodes a basic-helix-loop-helix PAS containing (bHLH-PAS) transcription factor required for embryonic tracheal development [30]–[32]. Here we describe a novel and essential role for *dys* during leg joint formation. We show that *dys* is absolutely required for tarsal joint formation while it is dispensable for proximal joints. Dys regulates the expression of the pro-apoptotic genes *rpr* and *head involution defective (hid)* and the Rho GTPase regulators *RhoGAP71E* and *RhoGEF2*. *dys* expression at the presumptive tarsal joints is controlled by a dedicated *cis*-regulatory module (CRM), directly regulated by Notch through binding of Su(H). In summary our results provide a molecular explanation of how Notch can regulate the formation of different types of joints along the leg PD axis.

## Results

### *dys* is expressed in the presumptive tarsal joints

**Figure 1:**
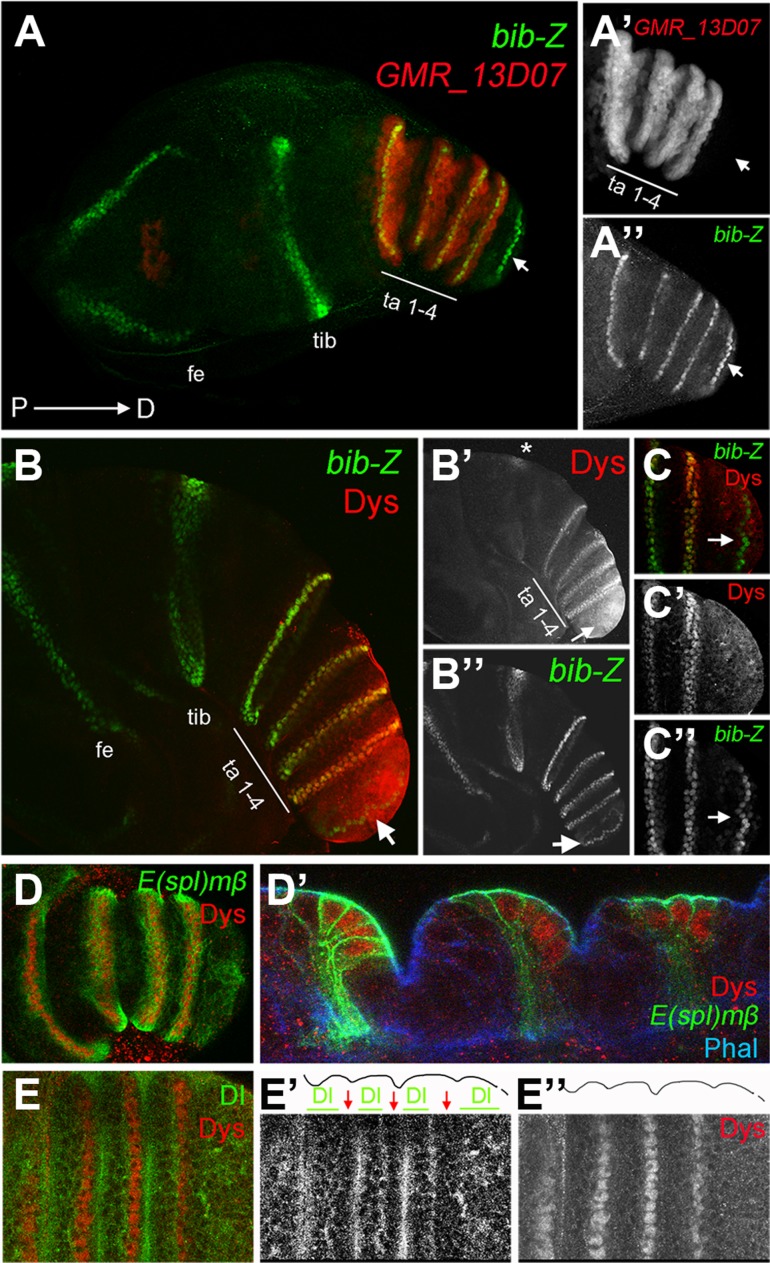
*dys* is expressed in the presumptive tarsal joints. (A) Activity of the Janelia line *GMR_13D07* that drives GFP expression (red) in the presumptive tarsal leg joints. Note that this line is not active in the most distal joint (arrow) or the proximal ones. *bib-Z* in green marks all the joints. Tarsal segments 1–4 (ta 1–4), tibia (tib) and femur (fe). (A′ and A″) Single channels for *GMR_13D07* and *bib-Z*, respectively. Distal tip of prepupal leg discs is to the right in all the panels. (B and C) Dys antibody staining (red) and *bib-Z* (green) expression in a prepupa leg disc. (C) Close view of the tip of the leg. Single channels for Dys (B′ and C′) and *bib-Z* (B″ and C″). Note that *dys* is expressed in the presumptive tarsal joints 1 to 4 (ta 1–4) and in an incomplete ring in the tibial/tarsal joint (asterisk), and that Dys is not expressed in the tarsal/pretarsal joint (arrow). (D) Dys (red) co-localize with *E(spl)mβ-CD2* (green) in the tarsal joints. (D′) Sagittal view of a prepupal leg disc epithelium stained with Phallodin (Phal) (blue), *E(spl)mβ-CD2* (green) and Dys (red). The joints between tarsal segments 1/2, 2/3 and 3/4 are shown. (E) Dys (red) is localized distal and adjacent to Dl domains (green) in the tarsal segments 1 to 4. Single channels for Dl (E′) and Dys (E″) are shown. The row of *dys*-expressing cells is marked with red arrows, while Dl domains are indicated with green bars in E′. Outlined of the leg is drawn in E′ and E″.

Proximal and distal leg joints are not only morphologically and functionally different, but also the mechanisms that sculpt them have evolved independently [18]–[22]. Since the Notch pathway is required for both types of joints, other factors should contribute to the differential development of distal *versus* proximal joints. To understand how the tarsal joints are genetically distinguished from the proximal joints, we have searched in the flylight database for CRMs exclusively active in rings in either tarsal or ‘true’ joints [33](see Materials and Methods). We identified two overlapping sequences within the *dysfusion* (*dys*) genomic locus that drove very similar or identical *GFP* reporter gene expression in concentric rings at the tarsomeres in third instar and prepupal leg imaginal discs (Figure 1A and Figure S1). We also detected *GFP* expression in distal rings in the antenna for these two CRMs (Figure S1). Using an available antibody for Dys [30], we confirmed that *dys* is expressed at the distal end of each tarsal segment in prepupal and third instar leg discs, with the exception of the boundary between the most distal tarsus and the pretarsus, which accounts for all the four tarsal joints (Figure 1B and 1C). We also detected an incomplete ring of expression at the distal tibia that was not reproduced by any of the two CRMs identified (Figure 1A and 1B).

The specification of joints is controlled by the local activation of the Notch pathway [11], [12], [14]. Notch downstream targets and its ligands Dl and Ser are expressed in complementary concentric rings along the PD axis of the leg. The Notch targets *bib* and *E(spl)mβ* are restricted to the distal end of each segment (joint domain), while the Notch ligands, Dl and Ser, are restricted to proximal adjacent cells (inter-joint domain) [11], [12], [14]. To confirm that *dys* expression is restricted to the presumptive joints we compared its expression with that of *bib*, *E(spl)mβ* and Dl. We found that *dys* coexpress with both joint markers at the distal-most cells of tarsal segments 1 to 4, while its expression is distal to Dl localization (Figure 1B–1E). In this manner, *dys* expression is restricted to Notch-responsive cells of the tarsal region, suggesting a potential role for *dys* in the development of tarsal joints downstream of Notch.

### *dys* is required for tarsal joint development and promotes epithelial fold formation

**Figure 2:**
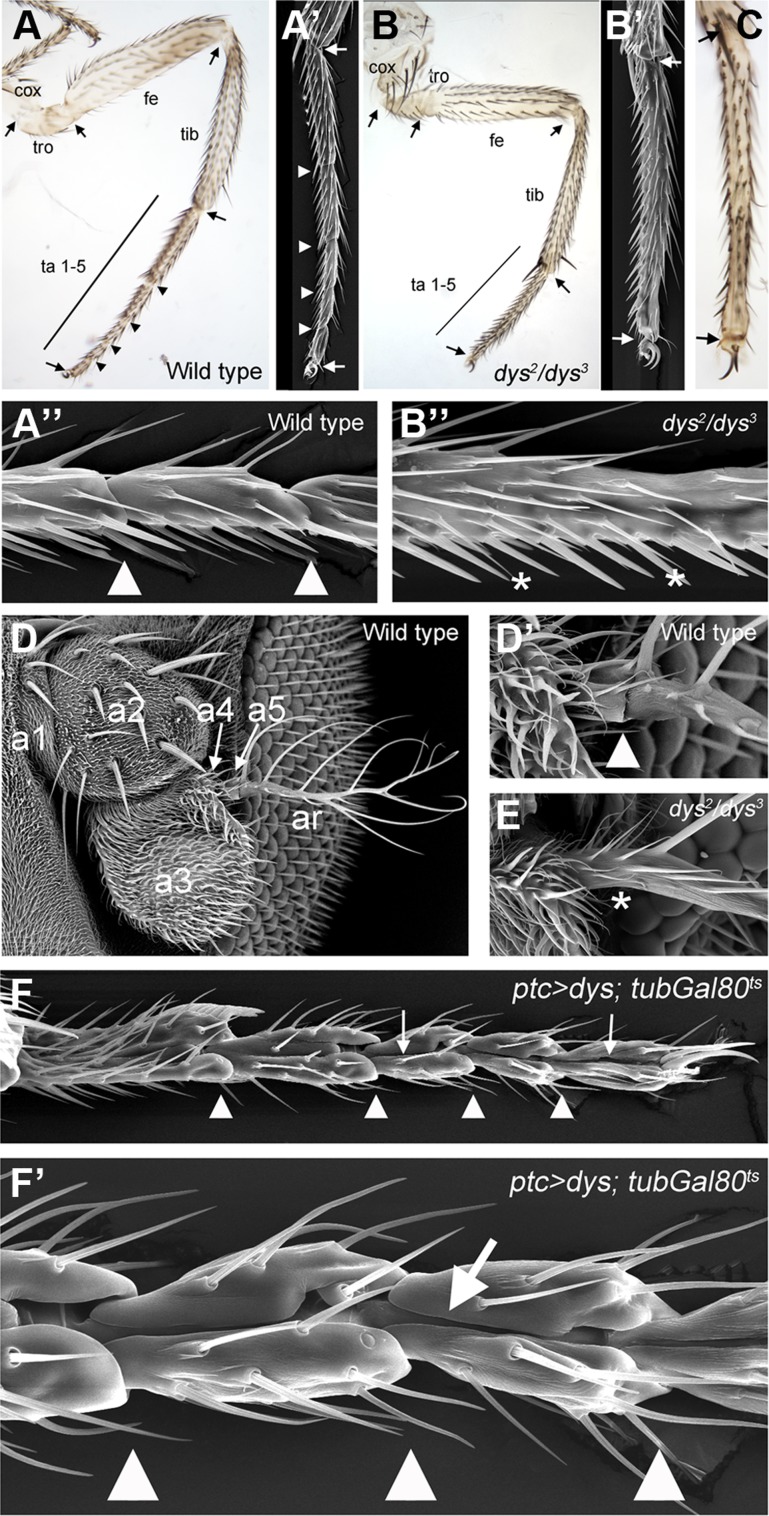
*dys* loss and gain of function phenotypes. (A) Adult leg of a wild type female, where the distal joints (arrowheads) and the “true” joints (arrows) are pointed out. (A′ and A″) Scanning Electron Microscopy (SEM) imaging of wild type legs. Close view of wild type tarsal joints is shown in A″. (B) Adult leg of a *dys^2^/dys^3^* mutant female. Note the complete absence of tarsal joints while the “true” joints are not affected (arrows). (B′ and B″) *dys^2^/dys^3^* mutant leg, and a close view of the tarsal region (B″). Note the lack of tarsal joints (asterisks). (C) *Dll-Gal4*; UAS-*dys*-RNAi female leg without tarsal joints. As in the *dys^2^/dys^3^* mutant leg, the tibial/tarsal and tarsal/pretarsal joints are not affected (arrows). (D) Wild type antenna, with antennal segments marked. (D′) Close view of the wild type a5/arista joint. (E) In a *dys^2^/dys^3^* mutant antenna the a5/arista joint is missing (asterisk). (F and F′) Male adult leg of a *ptc*-*Gal4*; UAS-*dys*; *tubGal80^ts^* fly. Ectopic expression of *dys* along the PD axis of the leg induced the formation of a fold (arrows). Arrowheads indicate normal position of tarsal joints.

To analyze in detail the role of *dys* during leg morphogenesis we first used a combination of two null *dys* alelles, *dys^2^/dys^3^*, which produces some escapers (less than 1% of the flies) (see Materials and Methods). All *dys^2^/dys^3^* adult flies display a complete absence of tarsal joint formation with a small shortening of the tarsal region (Figure 2A and 2B). Interestingly no defects were observed in most proximal joints (including the tibial/tarsal joint where a half-ring of *dys* expression is detected) or in the tarsus/pretarsus joint (Figure 2A, 2B and Figure S2A and S2B). In addition we detected the loss of the joint between the a5 and the arista in the antenna (Figure 2D and 2E). We also expressed a *dys*-RNAi construct that efficiently reduced Dys protein levels (Figure S2C). *dys* knockdown in the *Dll* domain (*Dll-Gal4*/UAS-*dys*-RNAi; distal tibia to claw) abolish joint formation in the tarsal segments without affecting the tibial/tarsal or tarsus/pretarsus joints just as described for *dys^2^/dys^3^* mutant legs (Figure 2C).

**Figure 3:**
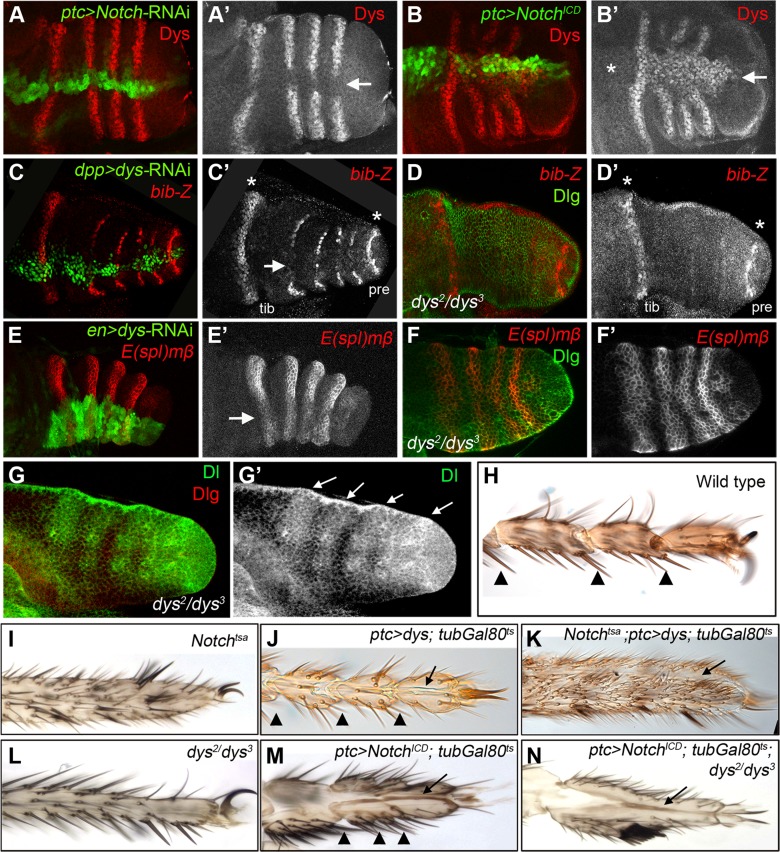
*dys* relationship with the Notch pathway. (A) *ptc*-Gal4, UAS-*GFP*; UAS-*Notch*-RNAi prepupal leg disc. Knockdown of Notch levels in the *ptc* domain (green and arrow in A′) downregulates *dys* expression (red and single channel in A′). (B) *ptc*-Gal4, UAS-*GFP*; UAS-*Notch^ICD^* prepupal leg disc. Notch pathway activation in the *ptc* domain (green and arrow in B′) induced *dys* expression (red and single channel in B′) in the tarsal segments but not in more proximal ones (asterisk). (C) *dpp-*Gal4. UAS-*GFP*; UAS-*dys*-RNAi prepupal leg disc. Dys knockdown in the *dpp* domain (green and arrow in C) downregulates *bib-Z* expression in the tarsal segments (red and single channel in C′). Note that *bib* expression in the tibial/tarsal (tib) and the tarsal/pretarsal (pre) joints remain unaffected (asterisks). (D) *dys^2^/dys^3^* mutant prepupal leg disc where *bib-Z* expression (red and single channel in D′) is downregulated in the tarsal segments while in the tibial/tarsal or the tarsal/pretarsal joints remain unaffected (asterisks). Discs-large (Dlg) is in green. (E) *en-*Gal4, UAS-*GFP*; UAS-*dys*-RNAi prepupal leg discs. Knockdown of Dys levels in the posterior compartment (green and arrow) slightly downregulated *E(spl)mβ-CD2* expression in the tarsal segments (red and single channel in E′). (F) *dys^2^/dys^3^* mutant prepupal leg disc. *E(spl)mβ-CD2* (red and single channel in F′) is still active in the presumptive tarsal joints. Dlg is in green. (G) Dl expression pattern (green and arrows) remains unaffected in *dys^2^/dys^3^* mutant prepupal leg. Dlg is in red and single channel for Dl is in (G′). (H–N) Distal adult legs of the following genotypes: (H) wild type, (I) *Notch^tsa^*,(J) *ptc*-Gal4; UAS-*dys*; *tubGal80^ts^*, (K) *Notch^tsa^*; *ptc*-Gal4, UAS-*dys*; *tubGal80^ts^*, (L) *dys^2^/dys^3^*, (M) *ptc*-Gal4; UAS-*Notch^ICD^*; *tubGal80^ts^* and (N) *ptc*-Gal4; UAS-*Notch^ICD^*; *tubGal80^ts^*; *dys^2^/dys^3^*. Normal tarsal joint formation is pointed out with arrowheads while ectopic folds along the PD axis are marked with arrows. Note the absence of tarsal joints in *Notch^tsa^* (I) and *dys^2^/dys^3^* (L) mutant legs, and the ectopic folds after *dys* (J) or *Notch^ICD^* (M) misexpression in a wild type background and in a *Notch^tsa^* (K) or *dys^2^/dys^3^* (N) mutant background.

To test whether *dys* is sufficient to induce joint-like structures in the leg, we ectopically expressed *dys* in an anterior row of cells along the PD axis of the leg disc using the *patched (ptc)*-*Gal4* line. We restricted *dys* ectopic expression to mid-third instar stage using the *Gal80^ts^* technique (see Material and Methods). *dys* misexpression induces the formation of cuticle folds along the PD axis of the leg that resembles ectopic joint formation (Figure 2F). These joint-like structures are more evident in the tarsal region, although we also detected ectopic folds in more proximal domains such as the tibia or the femur (Figure S2D). These phenotypes are very similar to the ones described for ectopic Notch pathway activation in the leg [12], [14] (see Figure 3M). Although we can not conclude that ectopic *dys* is able to induce the complete joint architecture, which would include the ball-and-socket structure, we were able to observe a phenotype that recapitulates some major aspects of joint formation such as indentation of the cuticle and fold formation. Taken together, our results suggest that *dys* is necessary for tarsal joint formation and sufficient to induce joint-like structures.

### *dys* is a target of the Notch pathway

*dys* expression is restricted to Notch responding cells in the presumptive tarsal leg joints, and its function is required for tarsal joint formation. To analyze the relationship between the Notch pathway and *dys*, we first tested if *dys* expression requires Notch activity. We found that Notch downregulation abolishes *dys* expression (*ptc-Gal4/*UAS*-Notch-*RNAi; Figure 3A), while Notch activation promotes *dys* expression (*ptc-Gal4/*UAS*-Notch^ICD^*; Figure 3B). Interestingly, although Notch activation was driven within the *ptc* domain along the entire PD axis of the leg, *dys* ectopic expression is restricted to the tarsal segments, suggesting that *dys* is a downstream target of Notch exclusively in the tarsal region. Next, we tested whether *dys* is required for the expression of two Notch targets, *bib* and *E(spl)mβ*. When Dys function is reduced or eliminated using *dys*-RNAi or the *dys^2^/dys^3^* allelic combination, respectively, we observed a strong downregulation of *bib-lacZ* expression in the tarsal segments, while *E(spl)mβ-CD2* is only slightly downregulated or remains unaffected (Figure 3C–3F.). Interestingly, the expression of *Dl* is not altered in *dys^2^/dys^3^* mutants (Figure 3G), indicating that neither *Dl* expression nor Notch activation require Dys function. In contrast, *bib-lacZ* expression does depend on Dys, although we do not know the basis of this regulation.

To study the functional relationships between the Notch pathway and *dys*, we first analyzed the ability of Dys to promote the formation of joint-like structures in a Notch mutant background. We induced *dys* ectopic expression in the *ptc* domain using the *tubGal80^ts^* technique in a hemizygous background for a *Notch* temperature-sensitive allele (*Notch^tsa^*) (see Material and Methods). *Notch^tsa^* mutants reared at 17°C (permissive temperature) and shifted to 29°C (restrictive temperature) at late third instar show a complete absence of tarsal joints (Figure 3I compared to 3H). As previously described, temporarily restricted *dys* misexpression in a wild type background induced cuticle invaginations in a joint-like fashion (Figure 3J). In *N^tsa^*; *ptc-Gal4*/UAS-*dys*; *tubGal80^ts^* flies shifted to 29°C at late third instar we found a uniform and continuous tarsal cuticle with no joints, characteristic of *Notch* mutants, and a fold running along the PD axis (compare Figure 3I and 3J with 3K). In the corresponding leg discs, ectopic *dys* expression induced the formation of an epithelial fold along the PD axis, both in wild type and in *N^tsa^* mutant backgrounds (compare Figure S2F and S2G with S2E). We also performed the reverse experiment, activating the Notch pathway in a *dys* null background. We found that in *dys^2^/dys^3^* mutant legs, forced expression of *N^ICD^* in the *ptc* domain still retains the capacity to make a fold even though endogenous tarsal joints are not formed (compare Figure 3L and 3M with 3N). This phenotype is very similar to that produced by the ectopic expression of *N^ICD^* in a wild type background (Figure 3M). This result suggests that forced Notch pathway activation in the absence of *dys* could be inducing other effector genes that play key roles in the formation of joint-like structures different from the tarsal ones. All together, these results demonstrate that *dys* is downstream of *Notch* during tarsal joint formation.

### Dys regulate genes implicated in leg joint morphogenesis

**Figure 4:**
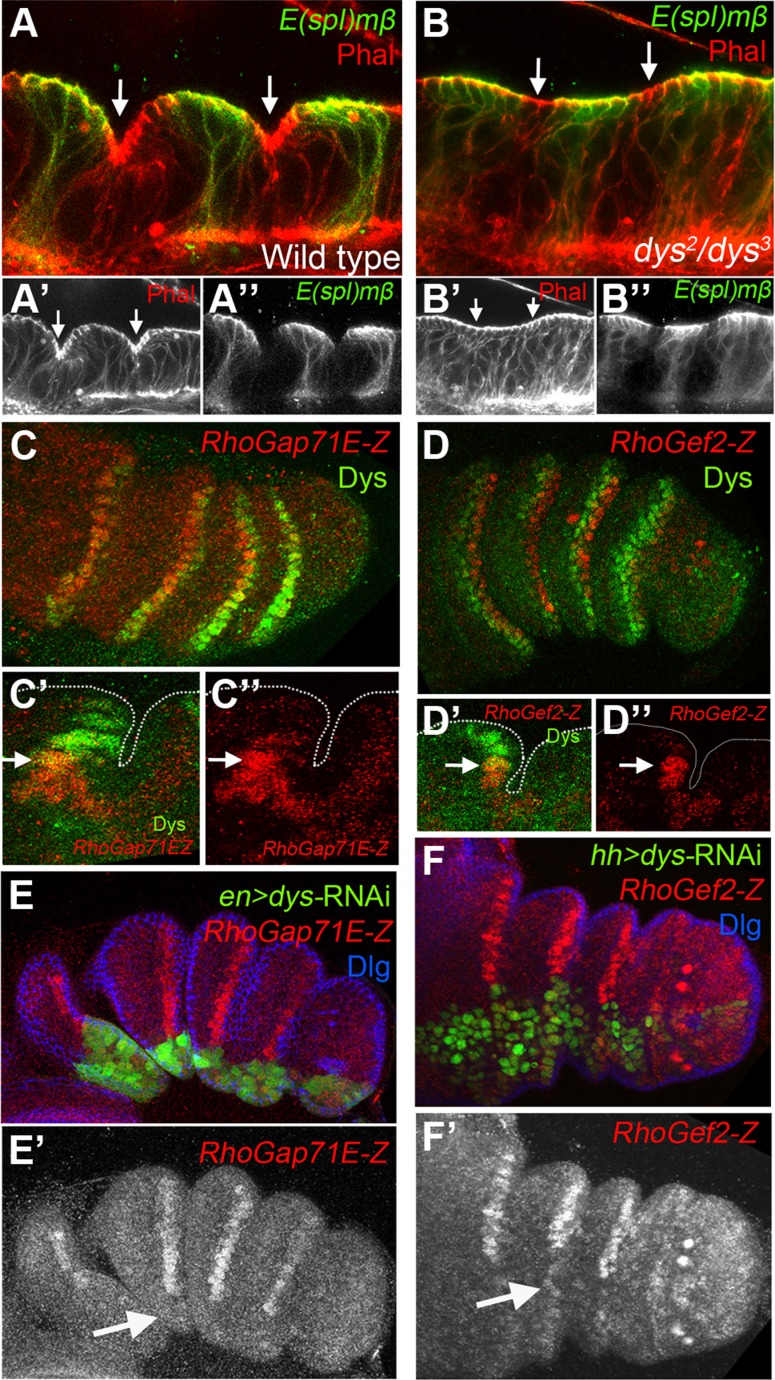
*dys* is required for fold formation and for the expression of *RhoGap71E* and *RhoGef2*. (A and B) Sagittal view of a wild type (A) and *dys^2^/dys^3^* (B) prepupal leg disc epithelium, stained with Phallodin (Phal) (red) and *E(spl)mβ-CD2* (green). The joints between tarsal segments 2/3 and 3/4 are shown in A (arrows), and the corresponding region in B (arrows). Note the apical constriction in cells immediately distal to the *E(spl)mβ-CD2* expression domain in the wild type leg and its absence in *dys^2^/dys^3^* mutant legs. Single channels for Phal (A′ and B′) and *E(spl)mβ-CD2* (B′ and B″) are displayed below. (C and D) *RhoGap71E-Z* (C, red) and *RhoGef2-Z* (D, red) expression compared to Dys (green). (C′ and D′) Sagittal view of a single joint where the last *dys* expressing cell is marked by an arrow. Note that both genes, *RhoGap71E-Z* and *RhoGef2-Z*, are expressed in a single row of Dys-positive cells and in a row of adjacent Dys-negative cells. (C″ and D″) Single channels for *RhoGap71E-Z* and *RhoGef2-Z*. (E) *en-Gal4*, UAS*-GFP*; UAS-*dys*-RNAi (green) and (F) *hh-Gal4*, UAS*-GFP*; UAS-*dys*-RNAi (green) downregulates *RhoGap71E-Z* and *RhoGef2-Z* expression (red, arrows), respectively. Dlg is in blue. Single channels for *RhoGap71E-Z* (E′) and *RhoGef2-Z* (F′) are displayed below.

**Figure 5:**
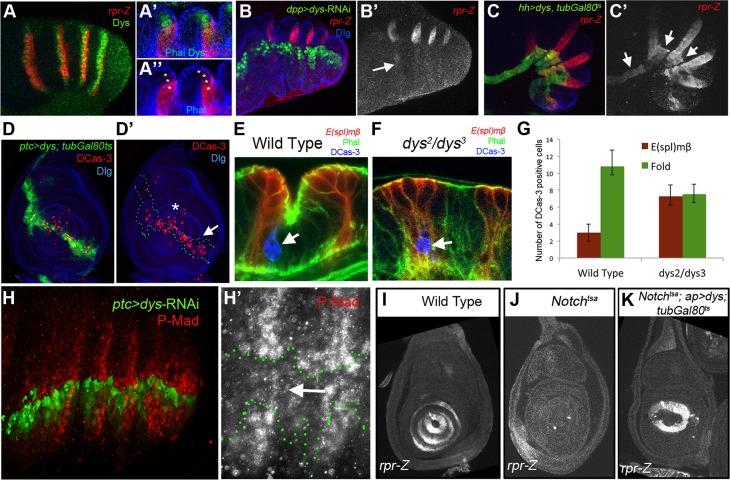
Dys regulates *rpr* expression and apoptosis. (A) *rpr-Z* (red) and *dys* (green) expression in prepupal leg. (A′ and A″) Close sagittal view of two tarsal joints (Phalloidin is in blue). Note that *dys* expression (green, asterisks) partially overlaps with *rpr-Z* (red), which extends a couple of cells distal to Dys. (B) *dpp-Gal4*, UAS*-GFP*; UAS-*dys*-RNAi (green) downregulates *rpr-Z* expression (red, and single channel in B′). *rpr-Z* downregulation is noted with an arrow in B′. (C) *hh-Gal4*, UAS*-GFP*; UAS-*dys; tubGal80^ts^* (green). Misexpression of *dys* for 24 hrs autonomously activates ectopic *rpr-Z* expression (red, and single channel in C′). Ectopic *rpr-Z* expression is pointed out with arrows. (D) *ptc-Gal4*, UAS*-GFP*; UAS-*dys; tubGal80^ts^* (green and green outline in D′). Misexpression of *dys* for 24 hrs in a third instar leg disc induces Caspase activity (DCas-3) cell autonomously (red, arrow). Dlg is in blue. (D′) Dlg and DCas-3 are shown. Asterisk marks the tip of the leg where endogenous high levels of DCas-3 are observed. (E–F) Close sagittal view of one tarsal joint in a wild type (E) and *dys^2^/dys^3^* mutant prepupal legs where the domains of *E(spl)mβ-CD2* (red) and the “fold” domain (between the two *E(spl)mβ-CD2*) are visible. Phal is in green and apoptotic cells are visualized by DCas-3 staining (blue, arrows). (G) Quantification of the number of apoptotic cells in the *E(spl)mβ*-CD2 and “fold” domains in wild type (n = 16 joints counted in 8 legs) and *dys^2^/dys^3^* mutant prepupae legs (n = 20 joints in 10 legs). Error bars represent the standard error of the mean. The number of apoptotic cells in the *E(spl)mβ-CD2* domain in *dys^2^/dys^3^* is significantly increased in mutant prepupal legs compared to wild type legs (p<0.05). A decrease in the number of apoptotic cells in the “fold” domain in *dys^2^/dys^3^* mutant prepupal legs can be observed, although is not statistical significant. (H) *ptc-Gal4-*UAS*-GFP*; UAS-*dys-*RNAi (green) prepupal leg. The activity of the Dpp pathway, visualized by P-Mad (red) is decreased after Dys knockdown in the *ptc* domain. (H′) A close up of the single P-Mad channel is shown with the *ptc* domain outlined in green. (I–K) *rpr-Z* expression in third instar leg imaginal discs of the following genotypes: (I) wild type, *rpr-Z* (J) *Notch^tsa^*; *ap*-*Gal4*, *rpr-Z* and (K) *Notch^tsa^*; *ap-Gal4*, *rpr-Z*/UAS-*dys*; *tubGal80^ts^* flies. (I) *rpr-Z* is expressed in rings in the presumptive tarsal joints. (J) *rpr-Z* fails to activate in *Notch^tsa^* larvae switched to 29°C for 24–48 hrs before dissection. (K) *Notch^tsa^*; *ap-Gal4*, *rpr-Z*/UAS-*dys*; *tubGal80^ts^* rescue *rpr-Z* expression after 24–48 hrs pulse of *dys* expression in the *ap* domain in a *Notch^tsa^* mutant background.

Epithelial cells at the presumptive joints undergo apical constriction and form characteristic folds that prefigure the future joint [22], [23]. The formation of epithelial folds involve cells immediately distal to the *E(spl)mβ-CD2* expression domain (Figure 4A). In contrast, in *dys* mutant prepupal legs these cells distal to the *E(spl)mβ-CD2* domain fail to reproduce these shape changes compared to control legs (compare Figure 4B and 4A). Two processes help sculpt the joint structure, namely JNK-mediated apoptosis driven by the pro-apoptotic gene *rpr* [20] and cell shape changes mediated by the Rho-family of GTPases [27]. The expression of *RhoGef2* and *RhoGap71E* is restricted to the tarsal segments where they are coexpressed with a single row of Dys-positive cells and are extended distally to a row of Dys-negative cells (Figure 4C and 4D). Downregulation of Dys levels with a *dys*-RNAi in the *engrailed* (*en*) or the *hedgehog (hh)* domains lead to compartment cell autonomous loss of *RhoGap71E* and *RhoGef2* expression, respectively (Figure 4E and 4F). Next, we compared the expression of *dys* with the pro-apoptotic genes *rpr* and *hid*. We found that the expression of these two genes in the prepupal leg discs is restricted to the distal end of each tarsal segments, where they are coexpressed with *dys* (Figure 5A, S3A) [20], [34]. Interestingly, as previously described for *RhoGap71E* and *RhoGef2*, we observed that *rpr* and *hid* are also expressed in a row of cells distal to *dys* expression (Figure 5A and S3A). Next we investigated if the expression of *rpr* and *hid* depends on *dys*. The downregulation of Dys levels in anterior cells along the PD axis with a *dpp*-*Gal4* line or in the posterior compartment with *en-Gal4*, strongly reduced or eliminated the expression of *rpr* and *hid*, respectively (Figure 5B and S3B). Furthermore, forced expression of *dys* in the posterior compartment for 24 hrs using the *tubGal80^ts^* technique is sufficient to promote cell autonomously *rpr* and *hid* expression in the inter-joint domain (Figure 5C and S3C). This ectopic activation of the pro-apoptotic genes *rpr* and *hid* is accompanied by an increase of cell death in larval and prepupal leg discs, as visualized by the presence of activated Caspase-3 (DCas-3) (Figure 5D and S3D). To test if the downregulation in the expression of the pro-apoptotic genes observed after reducing Dys levels is associated to a decrease in cell death, we compared the number of DCas-3 positive cells in wild type and *dys* mutant legs (see Material and Methods). To determine the precise location of the apoptotic cells during joint formation, we separately counted DCas-3 positive cells within the *E(spl)mβ* domain and at the gap between two *E(spl)mβ* domains (termed here as “fold” domain). In wild type prepupal legs we found a significant increase in the number of DCas-3 positive cells in the “fold” domain compared to the *E(spl)mβ* one, while in *dys* mutant legs such significant difference was not observed (Figure 5E–G). Interestingly, while the total number of apoptotic cells per segment was comparable between wild type (average = 13,8) and *dys* mutant legs (average = 14,8), the distribution of DCas-3-positive cells in the joint was altered. *dys* mutant legs have approximately the same number of apoptotic cells in the *E(spl)mβ* than in the “fold” domains (Figure 5E–G).

It has been proposed that sharp discontinuities of Dpp activity trigger JNK-mediated apoptosis through the activity of *rpr* [20], [28]. Therefore we first compared P-Mad (a readout of Dpp signaling) levels with *bib* expression, which, as we have shown previously, is coexpressed with Dys in the tarsal joints. P-Mad in prepupal legs forms a dorsal ring-like pattern with higher levels coincident with *bib*-expressing cells (Figure S3E). Next we tested if the absence of *rpr* expression observed in *dys*-RNAi legs could be due to a failure in the generation of Dpp activity borders. *ptc-Gal4*; UAS*-dys*-RNAi prepupae legs show downregulation of P-Mad levels, suggesting that *dys* is required for the correct formation of sharp Dpp activity boundaries at the tarsal joints (Figure 5H).

Our results suggest that Dys is a downstream effector of Notch signaling during tarsal joint development, which activates the expression of the pro-apoptotic genes *rpr* and *hid*. To confirm these results, we tested if in the absence of Notch activity Dys is still able to induce the transcription of *rpr*. We found that the depletion of Notch function for 24 to 48 hrs before dissection, using the *Notch^tsa^* allele, was sufficient to completely abolish *rpr-lacZ* expression in the leg disc (compare Figure 5I with 5J). As expected, *Notch^tsa^*; *ap*-*Gal4/*UAS*-dys*; *tubGal80^ts^* larvae switched to 29°C, 24 to 48 hrs before dissection show ectopic *rpr-lacZ* expression in the *ap* domain of the leg imaginal discs (a ring in tarsal segments 4 and part of 5) while no *rpr-lacZ* expression is detected in more proximal tarsal rings (Figure 5K). These results confirm our previous observations that indicate that Dys is epistatic to Notch during tarsal joint development. In summary, these experiments suggest that *dys* is necessary for the correct expression of the pro-apoptotic genes *rpr* and *hid* and the RhoGTPase modulators *RhoGap71E* and *RhoGef2*, being both key events during tarsal joint morphogenesis.

### Tango requirements during leg joint morphogenesis

**Figure 6:**
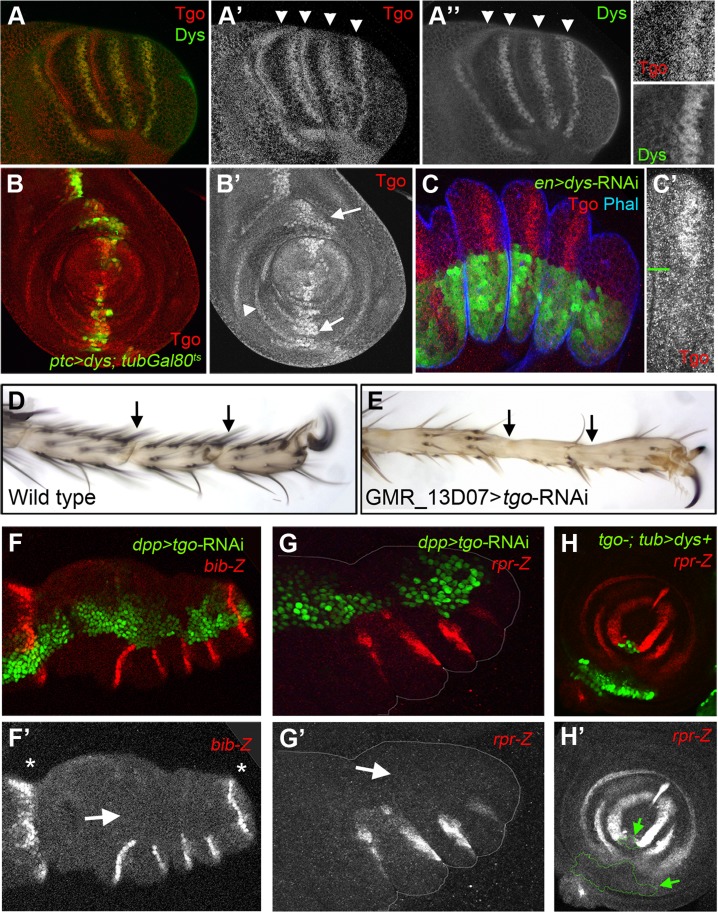
Tgo and Dys relationship during tarsal joint development. (A) Tgo (red) and Dys (green) co-localization in a prepupae leg. Note that Tgo nuclear protein localization coincides with that of Dys (arrowheads) in the presumptive tarsal joints. Single channels are displayed for Tgo (A′) and Dys (A″) and a close view of a single tarsal joint. (B) *ptc-Gal4*, UAS*-GFP*; UAS-*dys; tubGal80^ts^* (green) leg imaginal disc stained for Tgo (red and single channel in B″). After 24 hrs of *dys* misexpression, Tgo localizes to the nuclei in the *ptc* domain (green, arrows). *tgo* endogenous expression in tarsal rings is pointed out by arrowhead. (C) *en-Gal4-*UAS-*GFP*; UAS-*dys-*RNAi (green) prepupal leg disc stained for Tgo (red) and Phal (blue). After Dys knockdown, Tgo is no longer detected at the nucleus. (C′) A close view of a single tarsal segment is shown in which the border of compartment is marked with a green line. (D–E) View of the distal end of (D) wild type and (E) *GMR_13D07*-Gal4; UAS-*tgo*-RNAi adult legs. No tarsal joints are formed after Tgo knockdown in the presumptive tarsal joint domain (compare D with E; arrows indicate the position of the joints). (F–G) Knockdown of Tgo function in the *dpp* domain (green, *dpp-Gal4*, UAS*-GFP*; UAS-*tgo*-RNAi) strongly downregulates *bib-Z* (red in F) and *rpr-Z* (red in G) expression in prepupal leg discs. Note that no effect is observed in the tibial/tarsal or tarsal/pretarsal *bib-Z* expression (asterisks). Single channels for *bib-Z* (F′) and *rpr-Z* (G′) are displayed below. (H) *tgo^5^* mutant clones that also expressed *dys* under the *tub* promoter are marked by GFP (green) loose *rpr-lacZ* expression (red). (H′) Single channel for *rpr-Z* is displayed below, and the clone is outlined (arrows).

Tango (Tgo) is a bHLH-PAS transcription factor that is able to form heterodimers with multiple bHLH-PAS proteins including Spineless (Ss), Trachealess (Trh) and Dys [32], [35], [36]. In the absence of any of its partners, Tgo localizes in the cytoplasm while in the presence of a partner bHLH-PAS protein, Tgo and its companion form a complex that is translocated into the nucleus where it is functional [36]. During embryonic tracheal development, Tgo dimerizes with Dys to activate the transcription of tracheal fusion target genes [32]. To investigate if the Dys function dependency on Tgo also exists for tarsal joint formation, we analyzed Tgo protein localization and Tgo loss of function phenotypes during joint development. In prepupal leg imaginal discs, Tgo protein shows nuclear localization specifically where *dys* is expressed, at the presumptive tarsal leg joints (Figure 6A). Moreover, a 24 hr pulse of ectopic expression of *dys* (*ptc-Gal4*/UAS-*dys*; *tubGal80^ts^*) induced the nuclear localization of Tgo, while in cells where we reduced Dys levels (*en-Gal4*; UAS-*dys-*RNAi) Tgo fails to accumulate in the nucleus of presumptive tarsal joint cells (Figure 6B and 6C).

*tgo* loss of function phenotypes are characterized by fusions and deletions of tarsal segments without affecting proximal ones, phenotypes very similar to those of *ss* and *trh* null mutant flies [35], [37]. Since Tgo interacts with Ss to activate transcription, much of *tgo* tarsal leg defects were attributed to *Ss* phenotypes [35]. We decided to test if some of the *tgo* leg phenotypes described could be also due to Dys's inability to activate transcription in the absence of its partner. Tgo knockdown specifically at the presumptive tarsal joints using a *tgo*-RNAi driven by the *GMR_13D07*-*Gal4* line (see Material and Methods) disrupted joint formation, a phenotype very similar to *dys* mutant legs (compare Figure 6D with 6E and Figure 2B). As described for *dys* loss-of-function conditions, we found strong *bib-Z* downregulation in the presumptive tarsal joints in *dpp*-*Gal4*; UAS-*tgo*-RNAi flies, while the proximal or the most distal ones remain unaffected (Figure 6F). The same effect was observed in the expression of *rpr* and *RhoGap71E* after reducing Tgo activity (Figure 6G). All these phenotypes could be due to Dys's inability to activate transcription in the absence of Tgo. To test this possibility we have generated ectopic expression clones of *dys* in cells that are also mutants for *tgo* (see Material and Methods). As previously described above, *dys* ectopic expression in the leg activates *rpr* in a cell autonomous manner. As expected for a Tgo-obligated transcriptional co-activator, *dys* ectopic expression clones mutant for *tgo* are not able to induce the expression of *rpr* (Figure 6H). All together these results demonstrate that Dys functions with Tgo and both together regulate the expression of their target genes such as *rpr*.

### Binding of Su(H) to a *dys* 640 bp *cis*-regulatory module directly integrates Notch input to restrict *dys* expression to the joints

**Figure 7:**
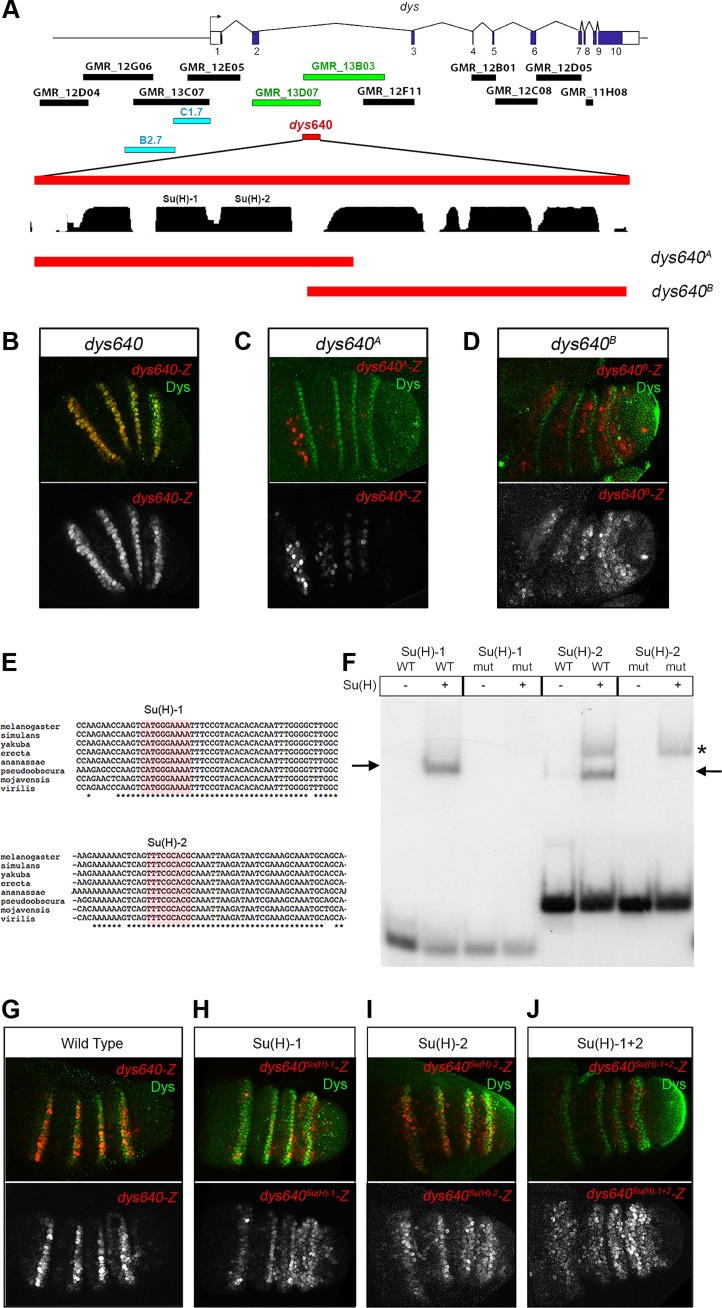
*dys* is a direct target of the Notch pathway. (A) Schematic representation of *dys cis*-regulatory region. Horizontal bars represent the DNA elements available in the Janelia database that maps around and within the *dys* gene. Blue bars correspond to the *dys*-CRMs identified by Jiang et al., 2010 [40] that drove reporter gene expression in fusion tracheal cells. Two Janelia lines (GMR_13D07 and GMR_13B03, green bars) drove reporter gene expression in the tarsal segments of the leg. The 640 bp overlapping sequence (*dys640*, red bar), DNA conservation between other *Drosophilds* and the two overlapping fragments (*dys640^A^* and *dys640^B^*) are also represented. (B–D) Prepupal leg discs stained for Dys (green) and for (B) *dys640-Z*, (C) *dys640^A^-Z* and (D) *dys640^B^-Z*. Note the perfect co-localization between *dys640-Z* and Dys (B). Single *lacZ* channels are displayed below. (E) DNA sequence of various *Drosophilid* species surrounding the two identified Su(H) binding sites (red shade) is shown. Asterisks mark perfect DNA conservation between species. Observe that both Su(H) sites are conserved (F) EMSA to assess binding of Su(H) to probes containing wild-type (WT) or mutated (mut) binding sites (see Material and Methods for sequences). Arrows indicate protein-DNA complexes, while asterisk indicate a non-specific band present in both wild type and mutant probe. (G–J) Prepupal leg discs stained for Dys (green) and for (G) *dys640-Z*, (H) *dys640^Su(H)-1^-Z*, (I) *dys640^Su(H)-2^-Z* and (J) *dys640^Su(H)-1+2^-Z*. All constructs have been inserted in the same genomic location and images were obtained keeping the confocal settings constant in the merge image. Single channels are displayed below, and for *dys640^Su(H)-1+2^–Z* the gain has been increase for visualization purposes.

We have screened 11 DNA fragments derived from the Janelia *Gal4* data base that cover the 5′ region and the introns of the *dys* genomic locus for expression in the leg imaginal disc [33]. Only two overlapping sequences, located between exons 2 and 3, GMR_13D07 and GMR_13B03, drive expression of a *GFP* reporter in a ring-like pattern that resembles *dys* expression in the leg (Figure 1A and S1). The overlapping sequence (640 bp long), cloned in a nuclear *lacZ* reporter vector (see Material and Methods), contains the information necessary to reproduce *dys* expression pattern in the tarsus (Figure 7B). We have previously shown that Notch acts upstream of *dys* and, in agreement with our genetic results, *dys640-lacZ* expression is disrupted in Notch knockdown prepupal leg discs (Figure S4A). Moreover, ectopic expression of *Notch^ICD^* activates *dys640-lacZ* expression, although this activation is restricted to the tarsus, just as described for *dys* endogenous expression (Figure S5B).

To gain further insights in the molecular regulation of *dys*, we divided the *dys640* CRM in two overlapping fragments and studied their expression using the same *lacZ* reporter construct (Figure 7A). The *dys640^A^-lacZ* fragment included two putative Su(H) binding sites conserved among different *Drosophila* species, and it is expressed weakly in patches of cells that do not overlap with *dys* expression (Figure 7C and 7E). Fragment *dys640^B^-lacZ* does not include these Su(H) sites and resulted in a weak but consistently extended expression of *lacZ* in the tarsal inter-joint domain, with low levels in *dys* expressing cells (compare Figure 7D with 7B). The two candidate binding sites were also tested for their ability to bind Su(H) in an electrophoretic mobility shift assay (EMSA) (Figure 7E and 7F). Su(H) binds to both putative sites and this binding was abolished when the sites were mutated (Figure 7F).

To further assess the contribution of the identified binding sites to *dys640-lacZ* expression, we mutated each individual binding site and the combination of Su(H)-1 and 2 *in vivo* in a transgenesis reporter assay. Mutation of either of these sites in isolation, *dys640^Su(H)-1^* and *dys640^Su(H)-2^*, significantly reduced but did not eliminate *lacZ* expression (Figure 7G–I). Interestingly we observed a slight derepression of *lacZ* signal in the inter-joint domain of all tarsal segments, being stronger in the fourth tarsal segment for the two constructs. The combined mutation of the two sites, *dys640^Su(H)-1+2^-lacZ*, resulted in an overall weaker expression than mutating each site separately and a derepression of *lacZ* in the inter-joints throughout the tarsal region and in the distal portion of the tibia (compare Figure 7J with 7G). Taken together, these results indicate that *dys* is a direct target of Notch through Su(H) binding to the *dys640* CRM. We propose that in the absence of Notch signaling, as in the tarsal inter-joint region, Su(H) binds to *dys640* CRM repressing *dys* expression. Conversely, Notch activation at the presumptive joint cells leads to loss of Su(H) repression, probably through a displacement of co-repressors and recruitment of co-activators, therefore converting Su(H) DNA-bound in a positive input to activate *dys* expression.

## Discussion

In this work we analyzed the function of the bHLH-PAS transcription factor Dys during leg joint morphogenesis. Dys has been previously characterized in *Drosophila* as a transcription factor involved in embryonic tracheal fusion and, as its mammalian ortholog Npas4/Nxf, forms a heterodimer *in vivo* with Tgo (Arnt) [30]–[32], [38]. We have found that *dys* is expressed in the presumptive tarsal joints, where it is required for tarsal joint development. In these cells, Dys regulates the expression of the pro-apoptotic genes *rpr* and *hid*, and the expression of the RhoGTPases modulators, *RhoGEf2* and *RhoGap71E*. When ectopically expressed, *dys* is able to induce some aspects of the morphogenetic program necessary for distal joint development such as fold formation and programmed cell death. As described for tracheal formation, this novel Dys function also depends on its obligated partner Tgo to activate the transcription of target genes. We also identified and characterized a dedicated *dys* CRM that regulates *dys* expression in the tarsal presumptive leg joints by Notch signaling through direct Su(H) binding. All these data place *dys* as a key player downstream of Notch, directing distal joint morphogenesis.

### Role of *dys* in tarsal joint formation

In a search for genes expressed exclusively either in distal or proximal leg joints we identified the gene *dys*. *dys* is coexpressed with *bib* and *E(spl)mβ* and it is distal to the Notch ligand Dl in four rings at the presumptive tarsal joints and in an incomplete ring at the presumptive tibial/tarsal joint. Legs mutant for *dys* do not develop tarsal joints and have instead a smooth continuous cuticle, without defects in other proximal joints or in the most distal one, the tarsus/pretarsus joint. In *dys* mutant prepupal leg discs the characteristic apical constriction of cells and the subsequent fold at the presumptive joint are lost. Conversely, ectopic expression of *dys* is able to induce ectopic folds along the leg that resemble joint-like structures, a phenotype very similar to those observed after misexpression of the activated form of Notch [12], [14]. Several experiments suggest that *dys* is a Notch target that is indispensable for tarsal joint development: (1) Notch directly regulates *dys* expression (see below). (2) The expression of the Notch targets *bib* and *rpr* absolutely require *dys* function, even when the Notch pathway is still active. (3) Dys is able to induce the expression of *rpr* and the formation of joint-like cuticle invaginations in the absence of Notch signaling. All these results place *dys* genetically downstream of Notch in the development of tarsal joints.

### *dys* is a new Notch-induced target in the distal leg

**Figure 8:**
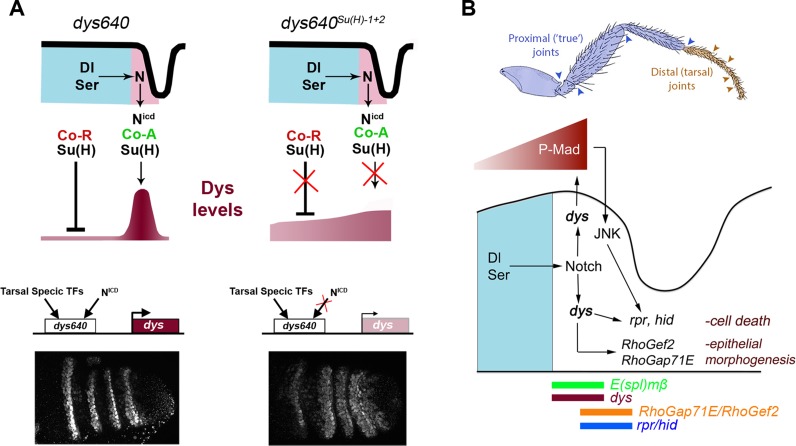
*dys* molecular regulation and model for tarsal joint development. (A) Schematic representation of a tarsal joint of a *dys640* wild type (left) and *dys640^Su(H)-1+2^* where the two Su(H) binding sites are mutated (right). See text for description. (B) Model for tarsal joint formation. Blue and brown arrowheads mark proximal and distal joints, respectively. In a tarsal joint, Notch ligands Dl and Ser (blue) activate the Notch pathway in the distal adjacent cells. Notch, in turn, activates *dys* expression, which regulates the high levels of P-Mad observed in the joint. Dys also controls the expression of the pro-apoptotic genes *rpr* and *hid* and the RhoGTPases modulators *RhoGef2* and *RhoGap71E*. The sharp boundary of Dpp signaling also regulates *rpr* expression via JNK pathway activation. The expression domains of *E(spl)mβ*, *dys*, *rpr*, *hid*, *RhoGef2* and *RhoGap71E* are indicated.

Our results show that *dys* expression at the presumptive tarsal segments is controlled by a dedicated CRM 640 bp long that integrates Notch signaling through direct Su(H) binding. This is, to our knowledge, the first characterized Notch direct target described for leg joint development. Interestingly, the mutation of the two identified Su(H) consensus sites, *dys640-lacZ^Su(H)-1+2^*, resulted in *lacZ* derepression in the inter-joint domain of the tarsal segments although at lower levels compared to normal signal observed in the presumptive joints in *dys640-lacZ* control legs. These results are in favor of the “default repression” model in which Su(H) associates with co-repressors in the absence of Notch signaling to repress target gene transcription [39] (Figure 8A). In the event of Notch activation, co-repressors are displaced by NICD, so Su(H) binding could lead to target gene transcription through the recruitment of co-activators. Therefore, *dys* fulfills the two predictions of the model to occur in the absence of Su(H) binding: (1) target genes will be derepressed and (2) their expression will be reduced in their normal expression domains. These two predictions can be validated in the *dys640^Su(H)1+2^* and *dys640^B^* gene reporter constructs, where Su(H) binding is compromised or lost. In both cases we observed a consistent derepression of *lacZ* expression in the inter-joint domain of the tarsal segments, although with weak levels. We also observed reduced *lacZ* expression at the presumptive joint domain in the *dys* CRM with the two Su(H) sites mutated or the *dys640^B^* reporter gene compared to the intact *dys640*. This characteristic is specially evident in the *dys640^B^* construct that lacks the two described Su(H) sites and probably others not identified in our analysis.

Enhancers are logic integrators of positive and negative inputs that drive precise temporal and spatial gene expression. In the case of *dys*, two previously identified CRMs drove reporter gene expression in all tracheal fusion cells in the embryo [40] (Figure 7A). For *dys* expression in the leg we have identified a different CRM that integrates Notch input and is only active in the tarsal presumptive joints. Although Notch pathway is active in rings along the PD axis of the leg, *dys* expression is restricted to the distal region, suggesting that other inputs are required to give *dys* its positional specificity along the PD axis. Thus, it is probable that tarsal transcription factors bind to *dys640* CRM to restrict its activity (Figure 8A). Some candidates are *bric à brac* (*bab*), *rotund* (*rn*) or *ss*, which are expressed in the four tarsal segments where *dys* is specifically active [41]–[43].

### Dys control of leg joint morphogenesis

Joint development involves a complex developmental program that leads to the formation of flexible structures connecting leg segments. At least two processes play key roles during tarsal joint development: epithelial morphogenesis controlled by the activity of RhoGTPases, and JNK-dependent programmed cell death [20], [27]. Several RhoGTPases modulators, RhoGaps and RhoGefs, which have a restricted expression pattern, are regulated by Notch and their downregulation affects joint formation [27]. Another important morphogenetic event is the formation of sharp Dpp signaling boundaries at the presumptive tarsal joints that triggers JNK-dependent localized cell death through *rpr* activation [20]. Interestingly P-Mad and *rpr* segmented expression depends on Notch activity, while ectopic formation of new Dpp signaling borders feedback to the system and activate downstream Notch targets such as *E(spl)mβ* [20]. Therefore, Notch controls tarsal joint morphogenesis in part through Dpp pathway and RhoGTPases regulation. Although a relationship between the Dpp pathway and the expression of RhoGTPases regulators has not been described, it is possible that these genes could also be regulated by sharp Dpp discontinuities, as is the case for *rpr*. Our results place *dys* directly downstream of Notch in the presumptive tarsal joints, regulating the Dpp pathway and the expression of *rpr*, *hid*, *RhoGef2* and *RhoGap71E* (Figure 8B). One interesting and unresolved question is how Dys can control the expression of *rpr*, *hid*, *RhoGap71E* and *RhoGef2* non-autonomously. We have described that these genes are expressed in a row of Dys-positive cells and in an additional Dys-negative distal row of cells (see Figure 4C, 4D, 5A, S3A and 8B). Interestingly, Dys downregulation blocks *rpr*, *hid*, *RhoGap71E* and *RhoGef2* expression both in *dys* expressing and non-expressing cells, while *dys* misexpression activate *rpr* and *hid* exclusively in a cell autonomous manner. Our results suggest that Dys could regulate *rpr* in two ways. The first one would be a direct activation by Dys and the second one an indirect effect through P-Mad and JNK pathway regulation that also regulates *rpr* [20]. Therefore, sharp Dpp discontinuities lead to JNK-mediated *rpr* expression while Notch activation in the presumptive tarsal joints activates *dys* expression that also regulates *rpr* cell autonomously. The cross talk between the Notch and Dpp pathways might be important to ensure a robust activation of *rpr* expression at the tarsal joints, and *dys* could be a key player in the communication between these pathways (Figure 8B). A similar mechanism to that described for *rpr* could regulate the expression of *hid*, *RhoGap71E* and *RhoGef2* in the presumptive tarsal joints.

It would be of interest to study if Dys controls the same subset of genes used for joint morphogenesis in other developmental contexts where it is expressed (e.g. embryonic tracheal fusion or leading edge cells) [30]. The role of Rho GTPases during tracheal development has been previously described [44], [45] but its implication in tracheal fusion and the possible role of Dys as a regulator of Rho GTPases in this process remains to be elucidated.

Another important observation is the discrepancy we observed between the expression of the pro-apoptotic genes and the number of DCas-3 positive cells. While in *dys* misexpression experiments we observed a positive correlation between the activation of *rpr*/*hid* and the increase of apoptotic cells, in *dys* mutant legs this relationship is more difficult to find. In wild type prepupal legs, the number of DCas-3 cells is significantly increased in cells distal to *E(spl)mβ* domain, while in *dys* mutants, this difference is not observed: there is a significant increase of apoptotic cells in the *E(spl)mβ* domain and a reduction, although not significant, in the “fold” domain. Therefore, our results suggest that in the absence of *dys* the apoptosis is not preferentially localized in any part of the joint, while in the presence of *dys*, this apoptosis is bent towards the cells distal to the *E(spl)mβ* domain. We propose that this imbalance of apoptosis is necessary for the correct formation of the tarsal joints.

### Proximal *vs* Distal joint morphogenesis

An important question in developmental biology is how a given signaling pathway is able to direct different morphogenetic programs. For example, the Dpp and the Sonic Hedgehog (Shh) pathways pattern the fly wing and the ventral neural tube, respectively, in a morphogene concentration-dependent manner, activating different sets of target genes (reviewed in [46]). In the case of the Notch pathway, target genes are regulated cell autonomously and depending on the cellular context a different set of downstream genes is regulated by Notch [15]. In the leg disc, the Notch pathway is similarly activated in 10 concentric rings along the PD axis that prefigure the future joints [11], [12], [14], [23]. Although all joints are homologous structures, distal joints differ from proximal or “true joints” not only in the absence of muscular attachments but also in their morphology, evolutionary origin and in the morphogenetic program that sculpt them [18], [20], [22]. Moreover, while each proximal joint has a unique morphology, all distal joints display the same ball and socket organization. Therefore, if Notch is absolutely required for all joints, the question arises of how Notch can be directing the formation of two very different yet homologous structures like distal and proximal joints. One possible scenario is that Notch controls joint morphogenesis through the activation of different sets of downstream effectors along the PD axis of the leg. Several Notch downstream target genes have been characterized to be required in all joints, such as *dAP-2* [16], while others are restricted to the proximal or “true joints”. Members of the *odd-skipped* family of zinc finger transcription factors are expressed in all joints of the leg except the tarsal joints and they might act redundantly to regulate the development of these proximal joints [21], [47]. Other genes are expressed exclusively in the tarsal joints like *tal*, *dpn* or *dys*. *dpn*, as *dys*, also encodes for a bHLH transcription factor expressed in the tarsal joints, although we did not find a phenotype in several *dpn* mutant allelic combinations or knocking down Dpn levels using a *dpn*-RNAi construct (data not shown) [26]. Interestingly, as it occurs in the embryonic trachea where Dys and another bHLH-PAS transcription factor, Trh, function in non-overlapping cells [30], in the leg these proteins are also present in distinct domains [37]. It would be interesting to study if the cross-regulation described in the trachea for Trh and Dys also exists in the leg.

Based on the expression and requirements for *dys* in the joints, we propose a model in which Notch directs the formation of the different joints by the PD-restricted activation of target genes such as *odd* in proximal joints and *dys* in the distal ones.

## Materials and Methods

### Drosophila strains

*dys^2^* and *dys^3^*, as well as the UAS-*dys* strains are described in [30], [31] and *GMR_13D07-* and *GMR_13B03-Gal4* and the rest of Janelia enhancer/GAL4 lines are described in the flylight database (http://flweb.janelia.org/cgi-bin/flew.cgi) [33] and are all publicly available at Bloomington Stock Center. *dpn* mutant alelles, *dpn^7^*, *dpn^6^* and *dpn^Def3D5^* were kindly provided by Antonio Baonza. The reporter lines *bib*-*lacZ* and *E(spl)-mβ*-CD2 [12] were used for assessing Notch pathway activation. To study the relation between *dys* and the Notch pathway, we used the UAS-*Notch^ICD^* [48], and UAS-*Notch-*RNAi lines [49]. The *Notch* thermosensitive mutant allele (*Notch^tsa^*) allowed us to knockdown *Notch* activity when the flies are shifted to the restrictive temperature (29°C) [50]. For loss- and gain-of-function experiments, we employed the *Gal4* lines *ptc-Gal4*, *dpp-Gal4*, *Dll-Gal4^MD212^*, *ap-Gal4*, *hh-Gal4 and en-Gal4* and the *tubGal80^ts^* allele, all-available at Bloomington Stock Center. We used the reporter lines *rpr-4kb-lacZ* (*rpr*-*lacZ*) [51], *hid*-*lacZ^W05014^* [52], *RhoGAP71E*- and *RhoGEF2*-*lacZ* (Bloomington Stock Center). The lines *dys-*, *tgo-* and *dpn-*RNAi are available at the Vienna Drosophila Resource Center (VDRC). RNAi knockdown experiments were performed on a UAS-*Dcr-2* background [53]. To generate *tgo* mutant clones we utilized the null allele *tgo^5^* (Bloomington) and the MARCM technique, which allowed us to simultaneously eliminate *tgo* function and express *dys* cell autonomously. The detailed genotype is: *yw hs-flp*, *tub-Gal4; UAS-dys; FRT 82B tubGal80/FRT 82B tgo^5^*. Loss of function clones were created by heat-shocking the larvae for 1 hour at 37°C 48 to 72 hrs after egg laying.

### Gain of function experiments

*dys* gain-of-function experiments were performed using the *Gal4-tubGal80^ts^* system, which allowed us temporal restriction of UAS-*dys* expression to mid-third instar stage. 24 hrs collection of *hh*-, *en*-, *ap-* or *ptc*-Gal4/UAS-*dys*; *tubGal80^ts^* flies were maintained at restrictive temperature (17°C) until mid-third instar stage, when the fly vials were shifted to the permissive temperature (29°C). Larva and prepupae were dissected between 24 to 48 hrs later.

### Immunostaining

Larval and prepupal leg discs were fixed and stained following standard procedures. As primary antibodies we used rabbit and mouse anti-βGal, rabbit anti-Dys (a gift from L. Jiang and S.T. Crews), rabbit anti-DCas-3 (cleaved *Drosophila* Dcp-1, Cell Signaling Technology), rabbit anti-P-Mad (kindly provided by G. Morata). Mouse anti-Dl, anti-Dlg and anti-Tgo are from Developmental Studies Hybridoma Bank, University of Iowa. TRITC-phalloidin and Phalloidin-Atto 647N were used to stain F-actin (Sigma Aldrich), and secondary antibodies were coupled to Red-X, FITC and Cy5 fluorocromes (Alexa Fluor Dyes, Invitrogen).

To determine the levels of cell death in *E(spl)-mβ* and “fold” domains, we have performed Z-stack imaging of wild type (n = 8 prepupae leg discs) and *dys^2^/dys^3^* mutants (n = 10 prepupae leg discs) and counted the number of D-Cas3 positive cells on each domain with the aid of the Fiji software. We selected for this analysis the joints between tarsal segments 2/3 and 3/4.

### Cloning of *dys* CRM in a *lacZ* reporter vector

The 640 bp overlapping DNA sequence between the *GMR_13D07* and *GMR_13B03* lines as well as the different mutant conditions were cloned in the HLz attB plasmid vector, which expresses a nuclear *lacZ* reporter under the control of the cloned sequence [6].

The primers used were the following for each reporter line (restriction sites are underlined and restriction enzyme used is noted in brackets):

***dys640*** **:**

Forward: 5′-cagtcctaggCCAAGCCGATGAGCCATTCCATACC-3′ (AvrII)

Reverse: 5′-cagtagatctCCACTCTGGAGCAAACCACACCGAA-3′ (BglII)

***dys640^A^*** **:**

Forward: 5′-cagtcctaggCCAAGCCGATGAGCCATTCCATACC-3′ (AvrII)

Reverse: 5′-cagtagatctTTCTGCTGATTTTCTTCTTTAGGTT-3′ (BglII)

***dys640^B^*** **:**

Forward: 5′-cagtcctaggCTCTCCATGGTTAAGCTCAGACTAA-3′ (AvrII)

Reverse: 5′-cagtagatctCCACTCTGGAGCAAACCACACCGAA-3′ (BglII)

Putative Su(H) binding sites were identified on the basis of a bioinformatics analysis combining data from the JASPAR CORE Insecta database (http://jaspar.genereg.net/) and the Target Explorer tool [54].

Mutagenesis of the Su(H) putative binding sites was performed using the QuikChange Site-Directed Mutagenesis Kit (Stratagene). We used the following primers: ***dys640^Su(H)-1^*** **:**

Forward: 5′-TCGATCCAAGAACCAAGTCcgagaccAATTTCCGTACACACACAA-3′

Reverse: 5′-TTGTGTGTGTACGGAAATTggtctcgGACTTGGTTCTTGGATCGA-3′ ***dys640^Su(H)-2^*** **:**

Forward: 5′-GGAGGAAGAAAAAACTCAGtggagacagCAAATTAAGATAATCG-3′ Reverse: 5′-CGATTATCTTAATTTGctgtctccaCTGAGTTTTTTCTTCCTCC-3′

*dys640*-*lacZ* reporter construct was inserted both in the 2R (51D) and 3R (86Fb) chromosomal locations. To allow proper comparison, all the *dys640*-*lacZ* versions (*dys640*-*lacZ*, *dys640^A^*-*lacZ*, *dys640^B^*-*lacZ*, *dys640^Su(H)-1^-lacZ*, *dys640^Su(H)-2^-lacZ* and *dys640^Su(H)-1+2^-lacZ*) were inserted in the same location. Confocal settings were kept constant when imaging wild type and mutant versions of *dys640*-*lacZ*, so *lacZ* expression levels are comparable between these conditions.

### Electrophoretic mobility shift assay

An incomplete form of Su(H), that bears the DNA binding domain [55] was translated *in vitro* using the TNT T7 Quick master MiX kit (Promega) and tested for binding with a series of labeled dsDNA probes. 50 ng of each sense oligonucleotide were labeled following standard procedures with γ-^32^P ATP, and then hybridized with the complementary “cold” oligonucleotide. Wild type and mutant probes, where nucleotides at consensus Su(H) binding site were mutated, were generated for the two identified Su(H) sites. The designed oligonucleotides were:

**Su(H)-1 WT**

Forward: 5′-CCAAGTCATGGGAAAATTTCC-3′

Reverse: 5′-GGAAATTTTCCCATGACTTGG-3′

**Su(H)-1 mut**

Forward: 5′-CCAAGTCcgagaccAATTTCC-3′

Reverse: 5′-GGAAATTggtctcgGACTTGG-3′

**Su(H)-2 WT**

Forward:

5′-GGAGGAAGAAAAAACTCAGTTTCGCACGCAAATTAAGATAATCG-3′

Reverse:

5′-CGATTATCTTAATTTGCGTGCGAAACTGAGTTTTTTCTTCCTCC-3′

**Su(H)-2 mut**

Forward: 5′-GGAGGAAGAAAAAACTCAGtggagacagCAAATTAAGATAATCG-3′

Reverse: 5′-CGATTATCTTAATTTGctgtctccaCTGAGTTTTTTCTTCCTCC-3′ Mutated Su(H) sites are noted with lower case letters.

### Scanning Electron Microscopy

Wild type and mutant adult flies were collected and their legs and heads dissected without any fixation and avoiding moisture prior to preparation for SEM. The preparation of the samples and Scanning Electron Microscopy was performed at the Microscopy Unit at Universidad Autónoma de Madrid.

## Supporting Information

Figure S1 Genomic localization of the *dys* Janelia lines. Schematic representation of *dys cis*-regulatory region (top). Black horizontal bars represent the DNA elements available in the Janelia database. Two Janelia lines (GMR_13D07 and GMR_13B03, green bars) drove *GFP* expression in the tarsal segments of the leg and in the antenna imaginal discs (green). Dll (red) and Hth (blue). Images are obtained from the flylight database.
(TIF)

Figure S2 dys gain and loss of function phenotypes. (A–B) SEM imaging close view of the tibial/tarsal joint in a (A) wild type and (B) *dys^2^/dys^3^* mutant legs. (C) *ptc-Gal4*, UAS*-GFP*; UAS-*dys*-RNAi (green) prepupal leg disc. *dys*-RNAi construct efficiently downregulates Dys protein levels (red and single channel in C′). Dlg is in blue. (D) Adult leg of a *ptc*-*Gal4*; UAS-*dys*; *tubGal80^ts^* fly. Temporally restricted ectopic expression of *dys* along the PD axis of the leg induced the formation of a fold in tarsal segments and the tibia (tib) (arrows). (E–G) Third instar leg imaginal discs stained with Dlg (red) of the following genotypes: (E) *ptc*-*Gal4*, UAS-*GFP*, (F) *ptc*-*Gal4*, UAS-*GFP*; UAS-*dys*; *tubGal80^ts^* and (G) *Notch^tsa^*; *ptc*-*Gal4*, UAS-*GFP*; UAS-*dys*; *tubGal80^ts^*. Cross-section of the leg imaginal disc and single channels for Dlg are also shown. Arrows indicate the presence of folds in the epithelium.
(TIF)

Figure S3 *dys* relation with the pro-apototic gene *hid* and P-Mad. (A) *hid-Z* (red) and *dys* (green) expression in a prepupal leg disc. (A′ and A″) Close view of two tarsal joints. Note that *hid* expression (red outline in A″) extends a couple of cells distal to Dys. (B) *en-Gal4*, UAS*-GFP*; UAS-*dys*-RNAi (green) downregulates *hid-Z* expression (red and single channel in B′, arrow). (C) *en-Gal4*, UAS*-GFP*; UAS-*dys; tubGal80^ts^* (green). Misexpression of *dys* for 24 hrs in the posterior compartment cell autonomously activates *hid-Z* expression (red and single channel in C′, arrow). (D) *en-Gal4*, UAS*-GFP*; UAS-*dys; tubGal80^ts^* (green). Misexpression of *dys* for 24 hrs induces caspase activity (DCas-3, red and single channel in D′) in the posterior compartment in a prepupa leg. The domain of *dys* misexpression is outlined in green. (E) P-Mad staining (red) and *bib-Z* (green) expression in a prepupal leg. Single channels for P-Mad (E′) and *bib-Z* (E″) are shown.
(TIF)

Figure S4 *dys*640 CRM is regulated by Notch signaling. (A) *ptc*-*Gal4*, UAS-*GFP*; UAS-*Notch*-RNAi; *tubGal80^ts^* (green). Notch knockdown downregulates *dys640-Z* expression (red and single channel) in a prepupal leg disc. (B) *ptc*-*Gal4*, UAS-*GFP*; UAS-*Notch^ICD^*; *tubGal80^ts^* (green). Notch pathway activation induces *dys640-Z* expression (red and single channel) in the tarsal segments in a third instar imaginal disc. Note that although Notch pathway activation is induced along the PD axis, *dys640-Z* expression is restricted to the distal domain of the leg.
(TIF)
