# Supplementary figures and images for: The bHLH-PAS Transcription Factor Dysfusion Regulates Tarsal Joint Formation in Response to Notch Activity during Drosophila Leg Development
Source: PLoS Genet. 2014 Oct 16;10(10):e1004621. doi: 10.1371/journal.pgen.1004621 (PMC4199481; doi:10.1371/journal.pgen.1004621)

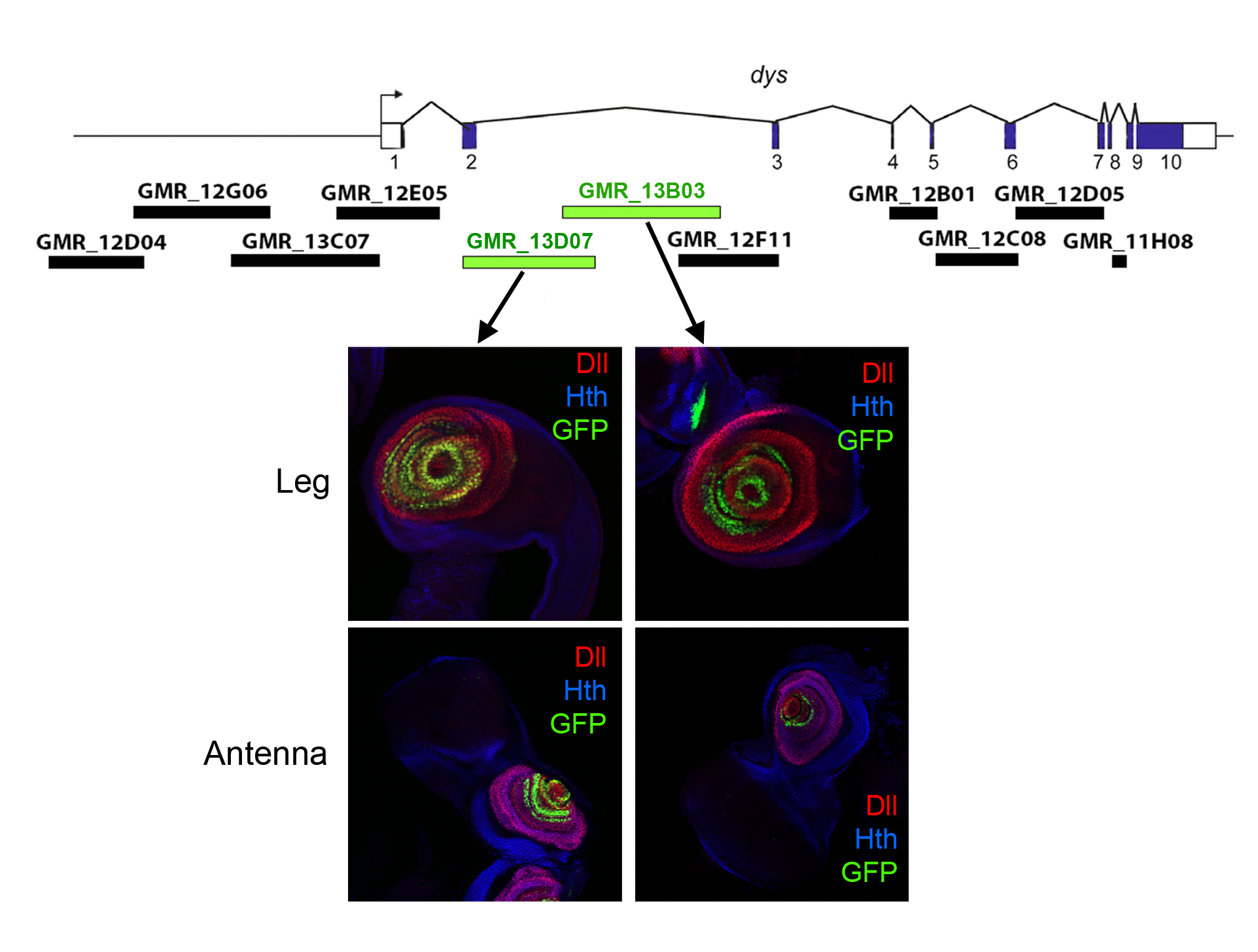

Supplement: Figure S1 — Genomic localization of the dys Janelia lines. Schematic representation of dys cis-regulatory region (top). Black horizontal bars represent the DNA elements available in the Janelia database. Two Janelia lines (GMR_13D07 and GMR_13B03, green bars) drove GFP expression in the tarsal segments of the leg and in the antenna imaginal discs (green). Dll (red) and Hth (blue). Images are obtained from the flylight database. (TIF) [file pgen.1004621.s001.tif]

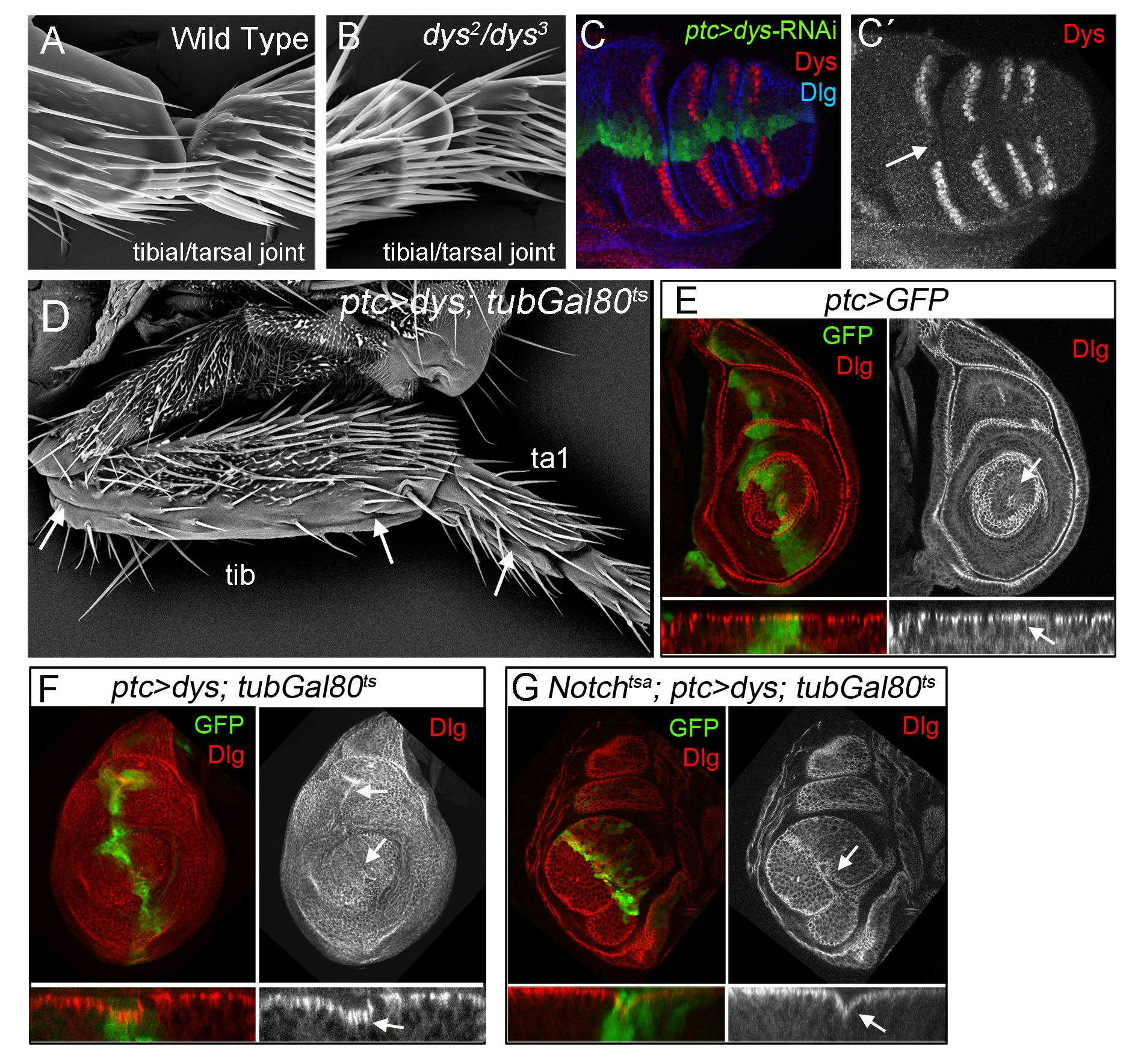

Supplement: Figure S2 — dys gain and loss of function phenotypes. (A–B) SEM imaging close view of the tibial/tarsal joint in a (A) wild type and (B) dys2/dys3 mutant legs. (C) ptc-Gal4, UAS-GFP; UAS-dys-RNAi (green) prepupal leg disc. dys-RNAi construct efficiently downregulates Dys protein levels (red and single channel in C′). Dlg is in blue. (D) Adult leg of a ptc-Gal4; UAS-dys; tubGal80ts fly. Temporally restricted ectopic expression of dys along the PD axis of the leg induced the formation of a fold in tarsal segments and the tibia (tib) (arrows). (E–G) Third instar leg imaginal discs stained with Dlg (red) of the following genotypes: (E) ptc-Gal4, UAS-GFP, (F) ptc-Gal4, UAS-GFP; UAS-dys; tubGal80ts and (G) Notchtsa; ptc-Gal4, UAS-GFP; UAS-dys; tubGal80ts. Cross-section of the leg imaginal disc and single channels for Dlg are also shown. Arrows indicate the presence of folds in the epithelium. (TIF) [file pgen.1004621.s002.tif]

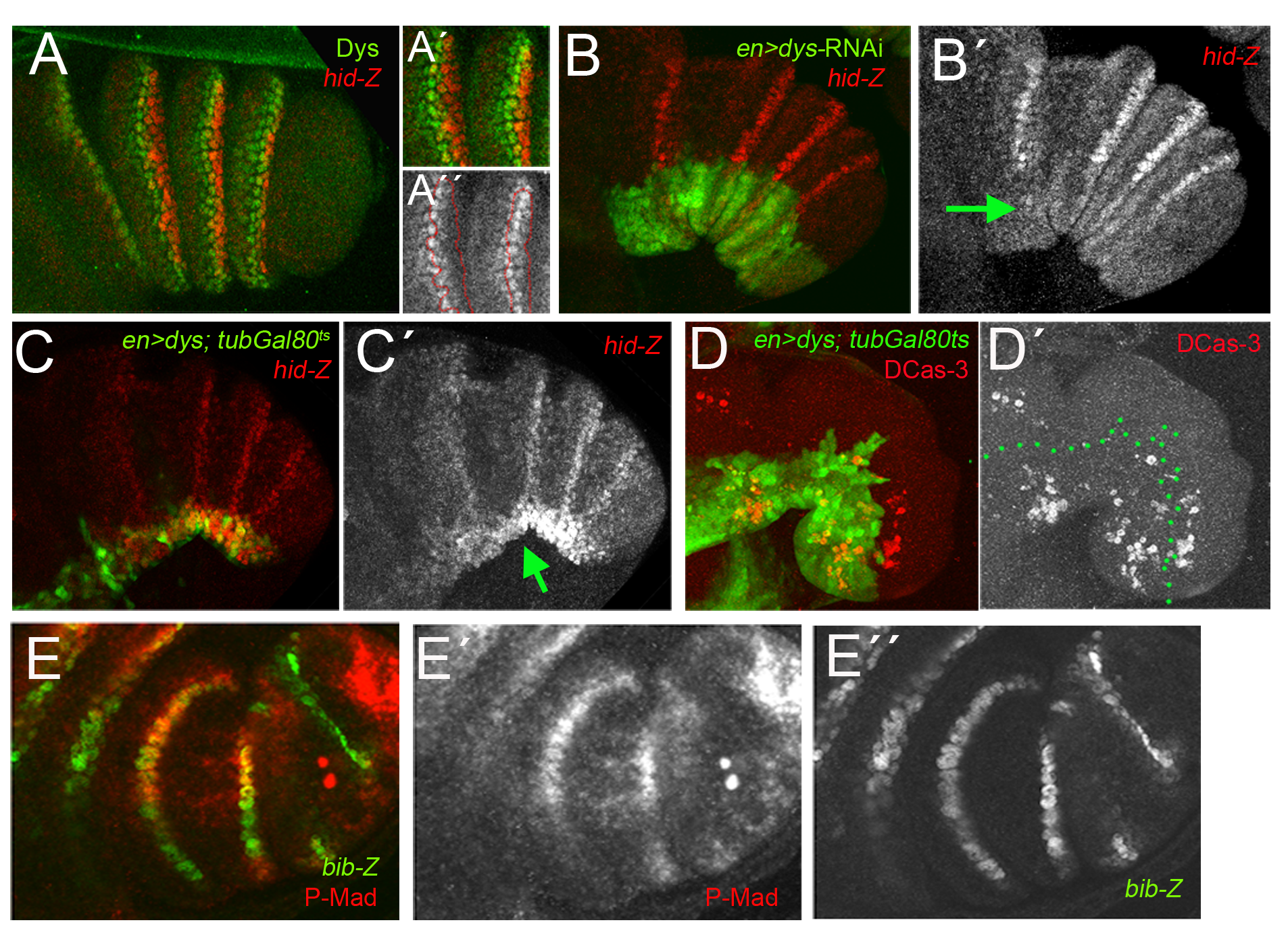

Supplement: Figure S3 — dys relation with the pro-apototic gene hid and P-Mad. (A) hid-Z (red) and dys (green) expression in a prepupal leg disc. (A′ and A″) Close view of two tarsal joints. Note that hid expression (red outline in A″) extends a couple of cells distal to Dys. (B) en-Gal4, UAS-GFP; UAS-dys-RNAi (green) downregulates hid-Z expression (red and single channel in B′, arrow). (C) en-Gal4, UAS-GFP; UAS-dys; tubGal80ts (green). Misexpression of dys for 24 hrs in the posterior compartment cell autonomously activates hid-Z expression (red and single channel in C′, arrow). (D) en-Gal4, UAS-GFP; UAS-dys; tubGal80ts (green). Misexpression of dys for 24 hrs induces caspase activity (DCas-3, red and single channel in D′) in the posterior compartment in a prepupa leg. The domain of dys misexpression is outlined in green. (E) P-Mad staining (red) and bib-Z (green) expression in a prepupal leg. Single channels for P-Mad (E′) and bib-Z (E″) are shown. (TIF) [file pgen.1004621.s003.tif]

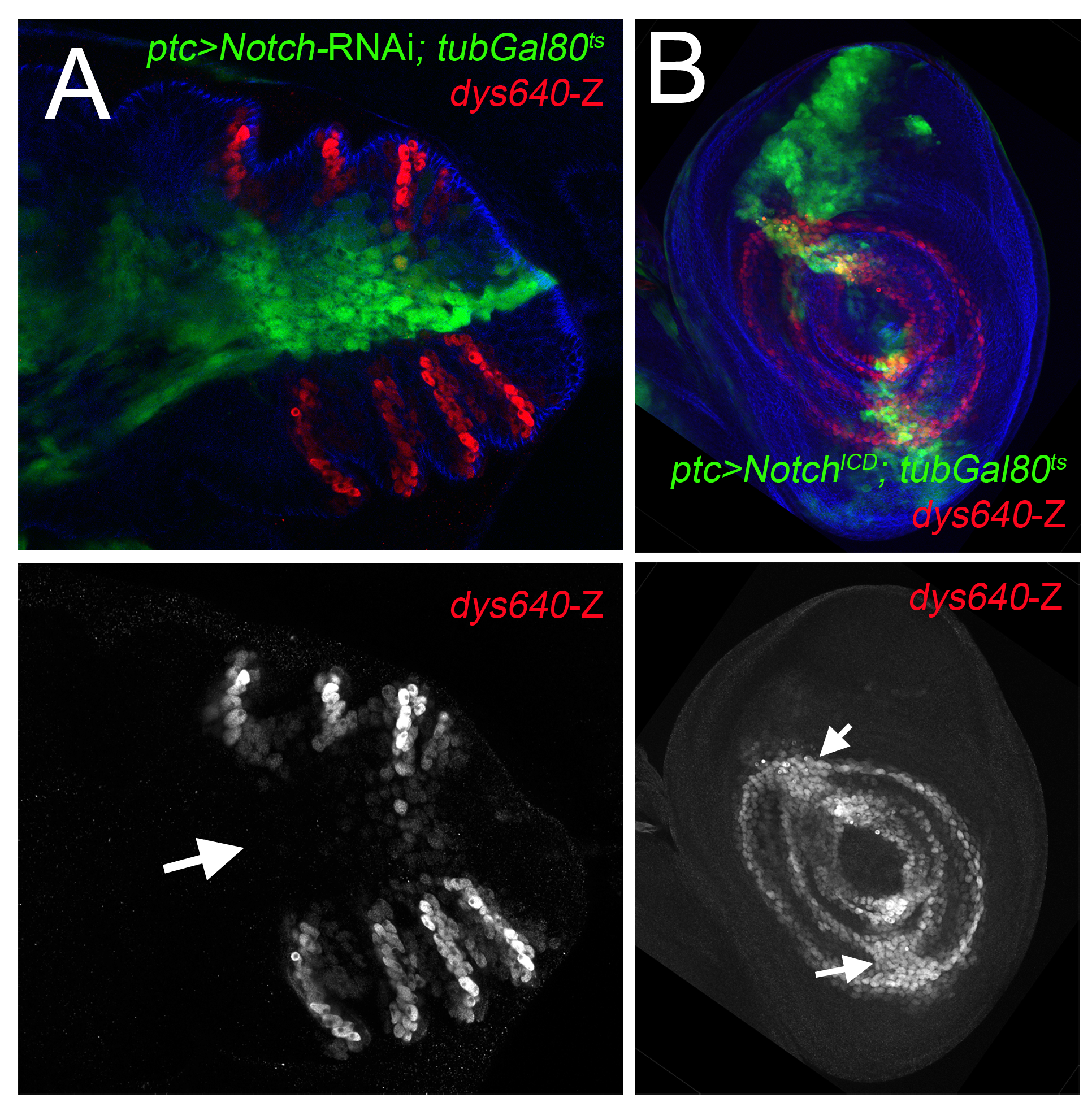

Supplement: Figure S4 — dys640 CRM is regulated by Notch signaling. (A) ptc-Gal4, UAS-GFP; UAS-Notch-RNAi; tubGal80ts (green). Notch knockdown downregulates dys640-Z expression (red and single channel) in a prepupal leg disc. (B) ptc-Gal4, UAS-GFP; UAS-NotchICD; tubGal80ts (green). Notch pathway activation induces dys640-Z expression (red and single channel) in the tarsal segments in a third instar imaginal disc. Note that although Notch pathway activation is induced along the PD axis, dys640-Z expression is restricted to the distal domain of the leg. (TIF) [file pgen.1004621.s004.tif]
